# Identification of novel therapeutic targets for Fuchs’ endothelial corneal dystrophy based on gene bioinformatics analysis

**DOI:** 10.1371/journal.pone.0264018

**Published:** 2022-03-03

**Authors:** Chao Liu, Zi-Qing Gao, Juan Li, Qi Zhou

**Affiliations:** Department of Ophthalmology, the First Affiliated Hospital of Bengbu Medical College, Bengbu, Anhui Province, China; Indiana University Purdue University at Indianapolis, UNITED STATES

## Abstract

Fuchs’ endothelial corneal dystrophy (FECD) is a disease where progressive visual impairment occurs by the thickening of the Descemet’s membrane and the gradual degeneration and loss of corneal endothelial cells. This study aimed to investigate the key changes in gene expression associated with FECD and explore potential biomarkers and new therapeutic strategies for FECD. To explore the potential therapeutic targets of FECD, we downloaded the gene expression dataset GSE171830 from the Gene Expression Omnibus (GEO) database. A total of 303 differentially expressed genes (DEGs) were identified by the limma package. The enriched Gene Ontology (GO) annotations and the Kyoto Encyclopedia of Genes and Genomes (KEGG) pathways of DEGs mostly included the extracellular matrix organization, collagen-containing extracellular matrix, and the structural constituents of the extracellular matrix. Fifteen hub genes from the most significant module were ascertained by Cytoscape. Both collagen-containing extracellular matrix and extracellular matrix hit to ANXA1, VCAN, GPC3, TNC, IGFBP7, MATN3, and SPARCL1 genes in the GO cellular components. Among these genes, the expression of SPARCL1 was down-regulated in the FECD samples, whereas the expression of GPC3, MATN3, IGFBP7, TNC, VCAN, and ANXA1 was up-regulated in the FECD samples. Gene set enrichment analysis (GSEA) plots showed that among the 20,937 genes, SPARCL1 played an important role in three pathways, cytokine-cytokine receptor interaction, the TGF-beta signaling pathway, and antigen processing and presentation. The top three pathways enriched by the GPC3, MATN3, IGFBP7, TNC, VCAN, and ANXA1 genes were those for cytokine-cytokine receptor interaction, TGF-beta signaling, and RIG-I-like receptor signaling. In conclusion, the DEGs identified here might assist clinicians in understanding the pathogenesis of FECD. Furthermore, these identified biomarkers might serve as potential therapeutic targets for the treatment of FECD.

## 1| Introduction

Fuchs’ endothelial corneal dystrophy (FECD) is characterized by a bilateral progressive loss of the corneal endothelial cells. The clinical signs of FECD include the formation of abnormal extracellular matrix material called guttata/guttae on the Descemet’s membrane [1]. FECD mainly occurs in the elderly over the age of 40 and shows a gender dichotomy. The Reykjavik Eye Study revealed FECD in 11% female and 7% male residents older than 55 years in Reykjavik, Iceland [2]; the Kumejima Study found a prevalence of FECD in 4.1% of the individuals who were above 40 years [3]. The female-to-male ratio of the occurrence of FECD was found to be 2.5–3:1 [4].

Currently, the treatment for FECD requires surgical treatments (e.g., Descemet stripping endothelial keratoplasty, Descemet membrane endothelial keratoplasty, Descemetorhexis without endothelial keratoplasty, Rho-associated kinase inhibitors, and cell-based approaches), and non-surgical and pharmacological treatments (e.g., topical application of hypertonic 5% sodium chloride eye drops or ointments). The annual preparation of donor tissue is difficult and has unacceptable risks, including loss of donor tissue, financial losses, and cancellation of surgery; therefore, these limit the acceptance of corneal transplantation in patients.

The pathophysiology of Fuchs’ endothelial dystrophy currently includes the following: 1. Genetics: mutations causing the early-onset of FECD have been exclusively linked to the α2 chain of collagen 8 (COL8A2) [5]. Extensive studies in recent years have led to the description of key genetic changes in the late-onset of FECD, which include genes for transcription factor 4 [6], transcription factor 8 [7], ATP/GTP-binding protein-like 1 [8], and solute carrier family 4 member 11 [9]. These findings may provide new and important insights into the pathogenesis of the disease. 2. Molecular pathomechanisms: endothelial cells in FECD generally appear to be under endoplasmic reticulum stress, showing markers of apoptosis [10], oxidative stress, and premature senescence [11, 12], and perform epithelial-mesenchymal transition as a self-defense mechanism. Loss of barrier function and/or pumping function occurs gradually, as shown by a reduction in the Naþ/Kþ ATPase expression [13]. However, the pathophysiological mechanisms of FECD are not fully elucidated. Therefore, more researchers aim to find new targets for the treatment of FECD.

Limma is an R and Bioconductor software package that performs large-scale analysis of gene differential expression, differential splicing, and expression profiles to obtain gene chips and high-throughput sequencing data. An original microarray dataset GSE171830 was downloaded; six FECD samples and six unaffected samples were analyzed in our study. The probe linear model and the affyPLM software package were used to control the data quality, and the gcrma software package was used to analyze the completeness and comparability of the dataset. Commonly changed DEGs were filtered from the integrated data. Additionally, the GO/KEGG pathway analysis, protein-protein interaction network construction, and CentiScape analysis were also performed to analyze the data. Finally, we screened 15hub genes, among which SPARCL1, GPC3, MATN3, IGFBP7, TNC, VCAN, and ANXA1were determined to be the key genes related to FECD.

## 2 | Materials and methods

### 2.1 | Access to public data

GEO (http://www.ncbi.nlm.nih.gov/geo) is a public functional genomics database that contains data on gene expression, chips, and microarrays [14]. The chip dataset GSE171830, used for expression profiling analysis, was downloaded from the GEO (GPL10558 Illumina HumanHT-12 V4.0 expression beadchip). According to the annotation information, the probes were converted into the corresponding gene symbols. The GSE171830 data contained six samples related to the FECD model and six unaffected samples used as a control group.

### 2.2 | Intragroup data repeatability test

The repeatability of the data within each group was verified by performing Pearson’s correlation test. The R programming language was used for statistical analysis and plotting graphs. We used R to plot a visual heat map of the correlations between all samples from the same dataset. Sample cluster analysis was used to test the repeatability of the intragroup data within the dataset. Principal component analysis (PCA) is a common method of sample clustering, which is usually used for gene expression, resequencing, diversity analysis, and other sample clustering based on the information from different variables.

### 2.3 | Identification of differentially expressed genes

We used the limma software package (Ritchie et al., 2015) to screen for differentially expressed genes between the FECD and unaffected groups, and further screened for differentially expressed genes by determining the fold change (log2 (fold change) > 2); the differences were considered to be significant at p-value < 0.01. We plotted heat maps and volcanoes and visualized differentially expressed genes by using the ggplot2 and heatmap packages in R.

### 2.4 | Functional analysis of DEGs

To determine the related pathways and functions for the regulation of the differentially expressed genes, we conducted Gene Ontology (GO) and Kyoto Encyclopedia of Genes and Genomes (KEGG) analyses. When p-value < 0.01, the GO term or KEGG pathway was identified as being significantly enriched by the genes. The gene function enrichment analysis was performed using the clusterProfiler software package in the R software. The GOplot and ggplot2 packages in the R software were used to plot the results of the GO and KEGG analyses, respectively.

### 2.5 | Protein-protein interaction (PPI) network construction and the analysis and mining of hub genes

The analysis of the functional interactions between proteins might provide insights into the mechanism of disease occurrence or development. We thus analyzed the protein interactions of different genes using the STRING database (http://string-db.org/) [15], and the maximum interaction score required was 0.9 (medium confidence) [16]. Cytoscape (version 3.6.1) was used to visualize the PPI networks [17]. Initially, we constructed the PPI network diagram in the Cytoscape software. Next, we determined the most important module of the network map by MCODE [18], a plug-in of Cytoscape. The criteria for the MCODE analysis comprised: degree cut-off = 2, MCODE scores > 5, max depth = 100, k-score = 2, and node score cut-off = 0.2. The hub genes were excavated for degrees ≥ 13 [19]. The clustering analysis of the hub genes was performed using Metascape (online analysis).

### 2.6 | Enrichment analysis by Gene Set Enrichment Analysis (GSEA)

We used the GSEA version 2.2.1 software for gene set enrichment analysis (Subramanian et al., 2005). The genes were divided into high and low expression groups according to the expression level of the key genes. We used the MSigDB database on the GSEA website to obtain the c2.cp.kegg.v6.0.symbols.gmtdataset. Then, we performed the enrichment analysis using the default weighted enrichment method and set 1,000 times the random combination.

## 3 | Results

### 3.1 Validation of the datasets

**Fig 1:**
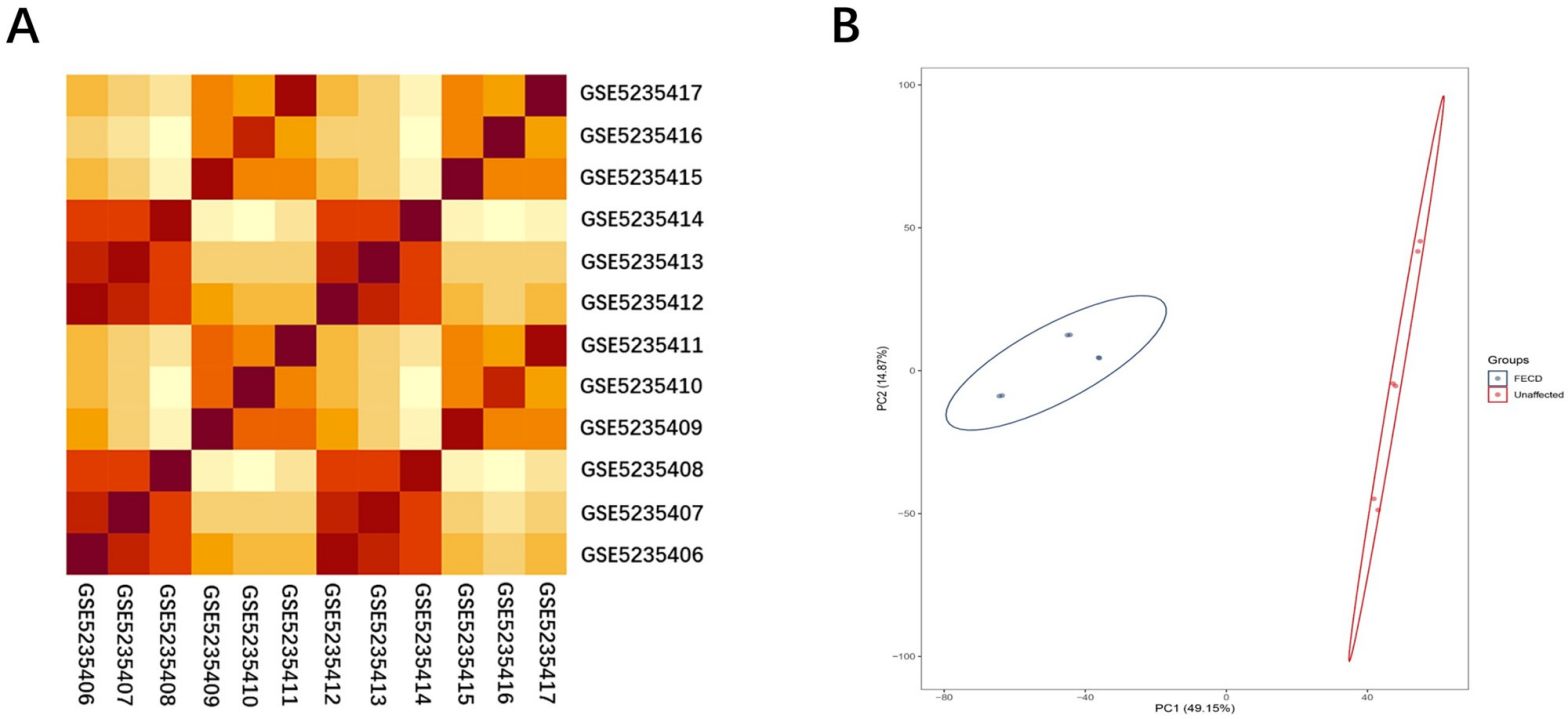
(A) Pearson’s correlation analysis of GSE171830 data set samples. The color reflects the strength of the correlation. When 0<correlation<1, there is a positive correlation. When -1<correlation<0, there is a negative correlation. The greater the absolute value of the number, the stronger the correlation. (B) The PCA of the GSE171830 data set samples. In the figure, principal component 1 (PC1) and principal component 2 (PC2) are used as the X-axis and Y-axis, respectively, to draw a scatter plot, where each point represents a sample. In such a PCA diagram, the greater the distance between two samples, the greater the difference in gene expression patterns between the two samples.

We performed Pearson’s correlation test and PCA to validate the repeatability of the intragroup data. Results of the Pearson’s correlation test showed that there was a strong correlation among the samples within the FECD group, as well as within the unaffected group for GSE171830 (Fig 1A). Results of the PCA showed that the repeatability of the intragroup data of the GSE171830 dataset was acceptable. The distance between samples in the unaffected group, as well as in the FECD group, was very short (Fig 1B).

### 3.2 | DEGs identified between FECD-affected and unaffected cells

**Fig 2:**
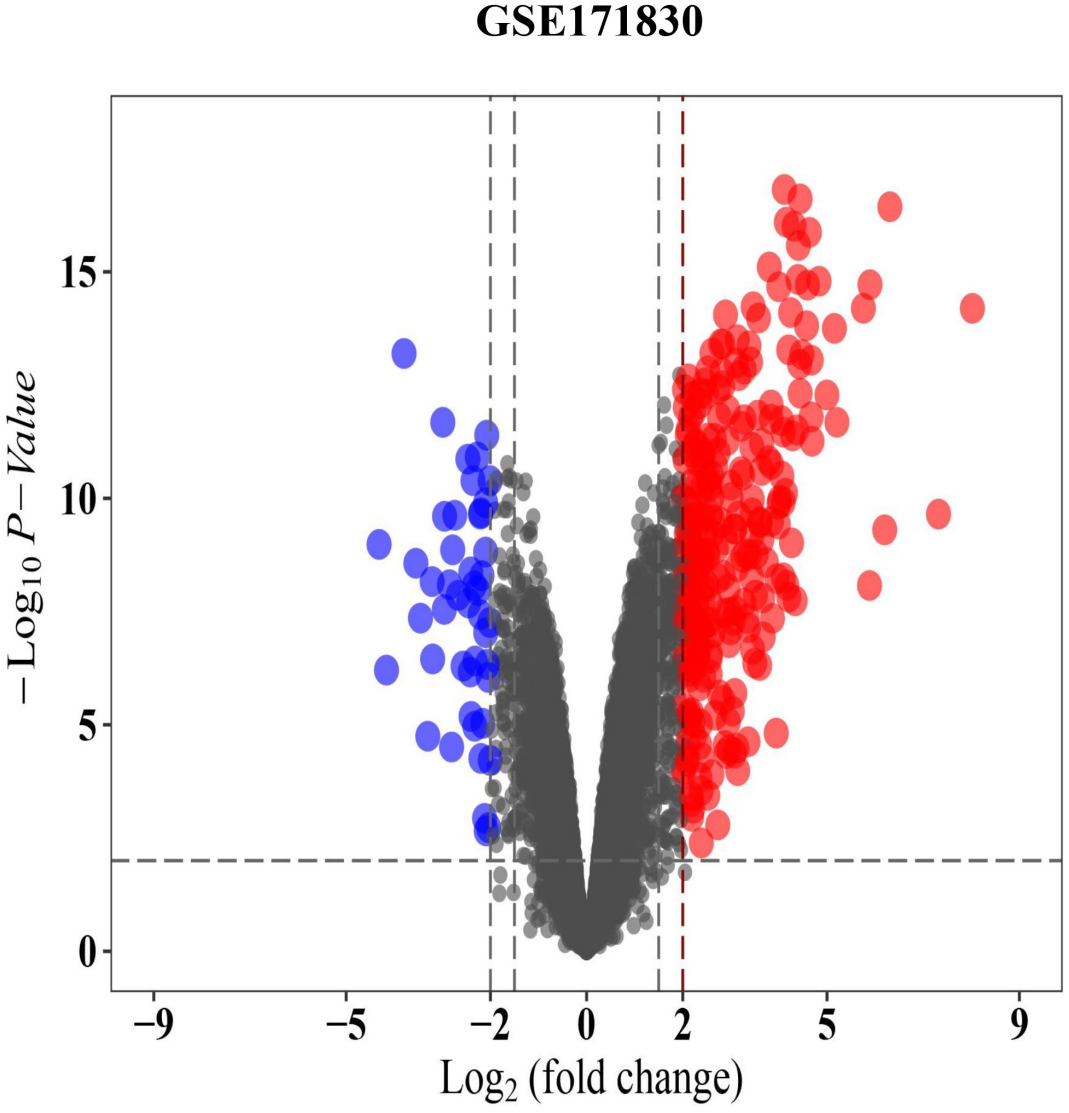
Differentially expressed gene analysis of GSE171830 datasets. The volcanic map of differential expression. In the GSE171830 data set, we identified 303 differentially expressed genes, including 257 upregulated and 46 downregulated genes.

**Fig 3:**
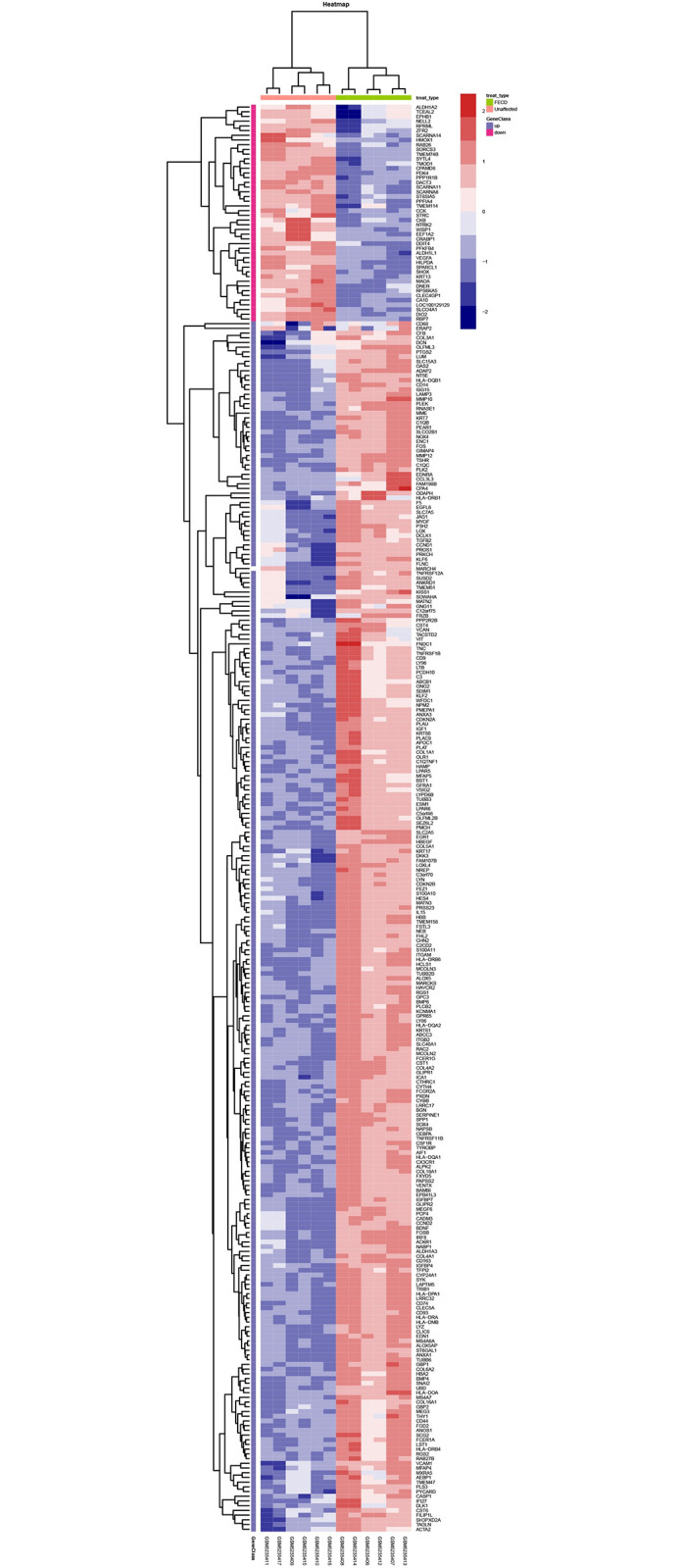
The heat map of the up-regulated genes and the down-regulated genes, blue indicates a relatively low expression and red indicates a relatively high expression.

We downloaded the gene expression profile GSE171830 from the GEO database and used the limma software package to screen for differentially expressed genes from the FECD-affected and unaffected corneal endothelium-Descemet’s membrane (CE-DM) of *Homosapiens*. We used a volcano plot (Fig 2) and a heat map (Fig 3) to display all the DEGs, with the upregulated and the downregulated genes, respectively.

### 3.3 | Functional annotation of DEGs by the GO and KEGG analyses

**Fig 4:**
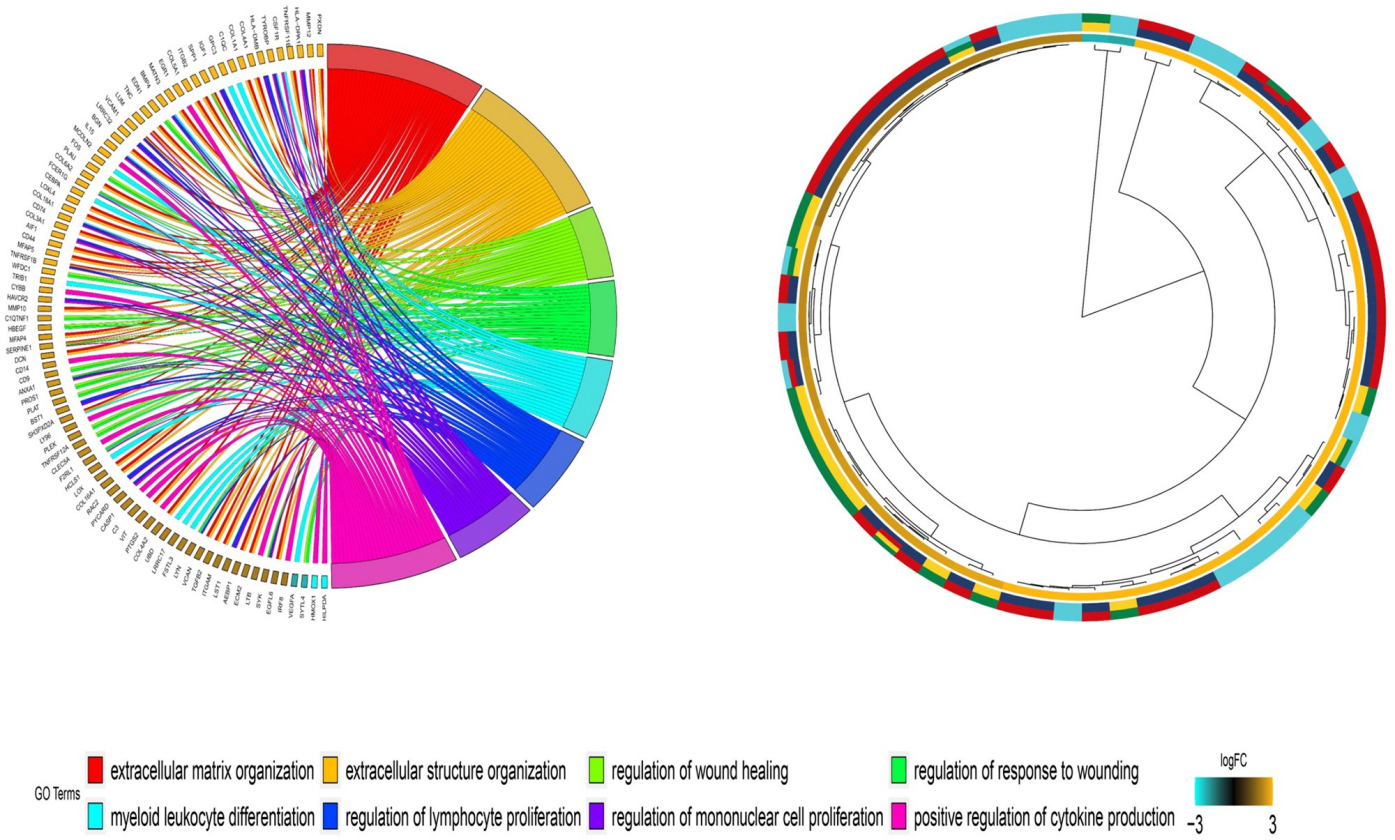
Biological Processes (BP) of the Gene Ontology (GO) analysis of upregulated and downregulated diferentially expressed genes.

**Fig 5:**
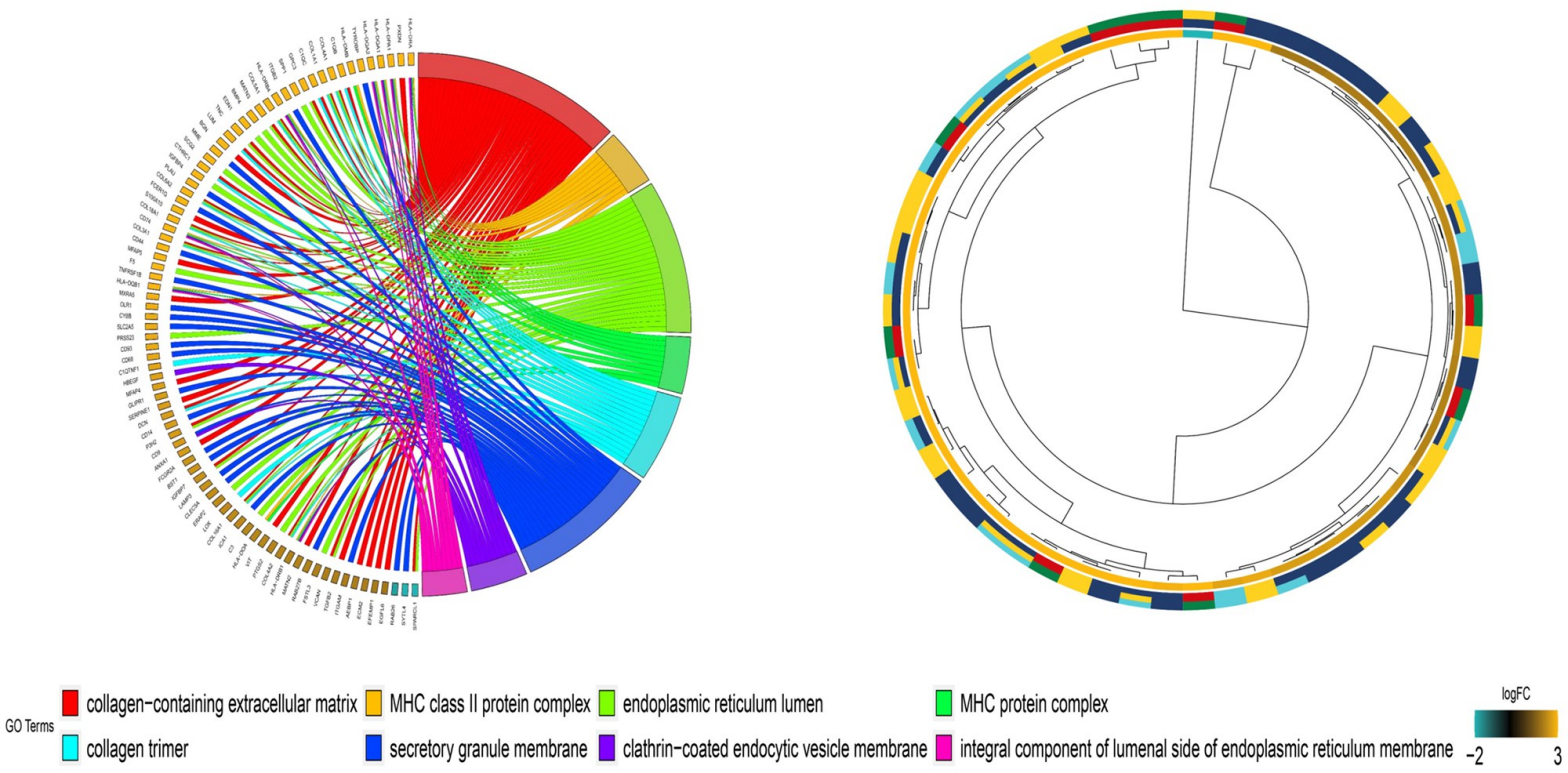
Cellular Component (CC) of the Gene Ontology (GO) analysis of upregulated and downregulated diferentially expressed genes.

**Fig 6:**
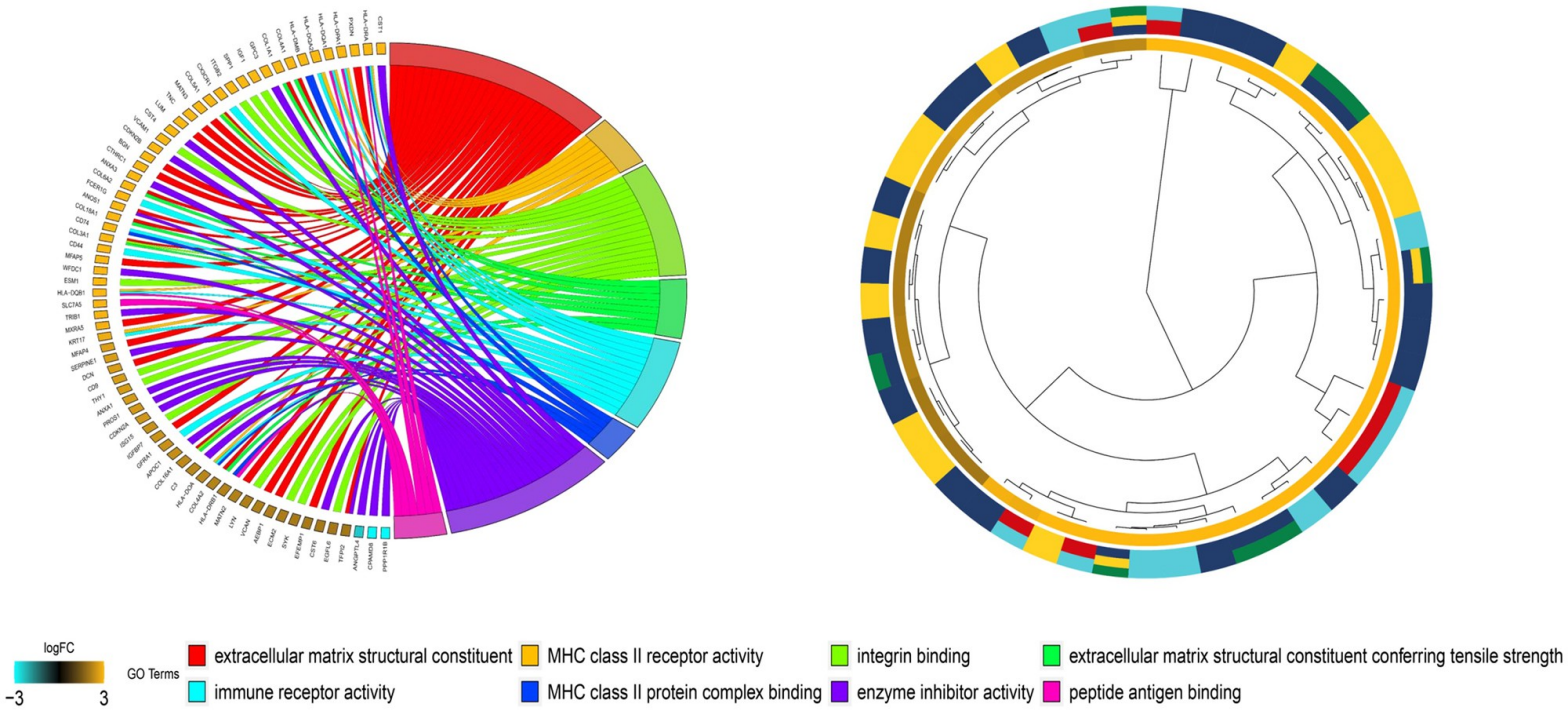
Molecular Functions (MF) of the Gene Ontology (GO) analysis of upregulated and downregulated diferentially expressed genes.

**Fig 7:**
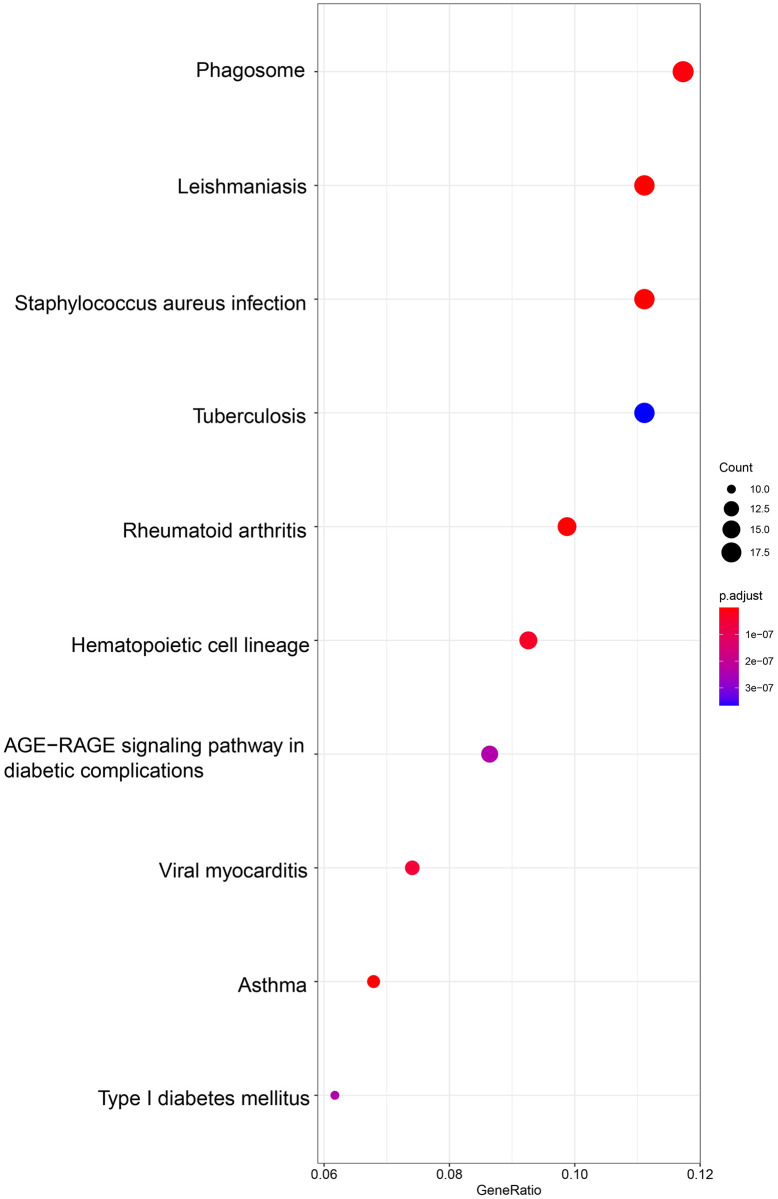
The KEGG pathway analysis of up-regulated and down-regulated diferentially expressed genes. Only enriched the top 10 signaling pathways, each of which had significant differences.

The differentially expressed genes were functionally analyzed by the clusterProfiler software package in the R software. For the GO functional categories, the three biological processes with the highest enrichment degrees were: 1) positive regulation of extracellular matrix organization, 2) extracellular structure organization, and 3) regulation of wound healing (Fig 4). The target genes were significantly clustered into items, including the collagen-containing extracellular matrix, MHC class II protein complex, and endoplasmic reticulum lumen in the cellular component (CC) process (Fig 5). The extracellular matrix structural constituent, MHC class II receptor activity, integrin binding, and other vital molecular functions (MF) were significantly related to these genes (p-value < 0.01) (Fig 6). In the KEGG functional enrichment analysis (Fig 7 and Table 1), we enriched only the top 10 signaling pathways, each of which had significant differences.

**Table 1:** KEGG enrichment analysis using Metascape.

ID	Description	GeneRatio	p.adjust	Count
hsa05140	Leishmaniasis	18/162	1.37E-12	18
hsa05150	Staphylococcus aureus infection	18/162	4.14E-11	18
hsa05310	Asthma	11/162	6.68E-10	11
hsa05323	Rheumatoid arthritis	16/162	1.80E-09	16
hsa04145	Phagosome	19/162	6.14E-09	19
hsa04640	Hematopoietic cell lineage	15/162	3.29E-08	15
hsa05416	Viral myocarditis	12/162	5.45E-08	12
hsa04940	Type I diabetes mellitus	10/162	2.37E-07	10
hsa04933	AGE-RAGE signaling pathway in diabetic complications	14/162	2.37E-07	14
hsa05152	Tuberculosis	18/162	3.66E-07	18

### 3.4 | PPI network construction, module analysis, and hub gene selection and analysis

**Fig 8:**
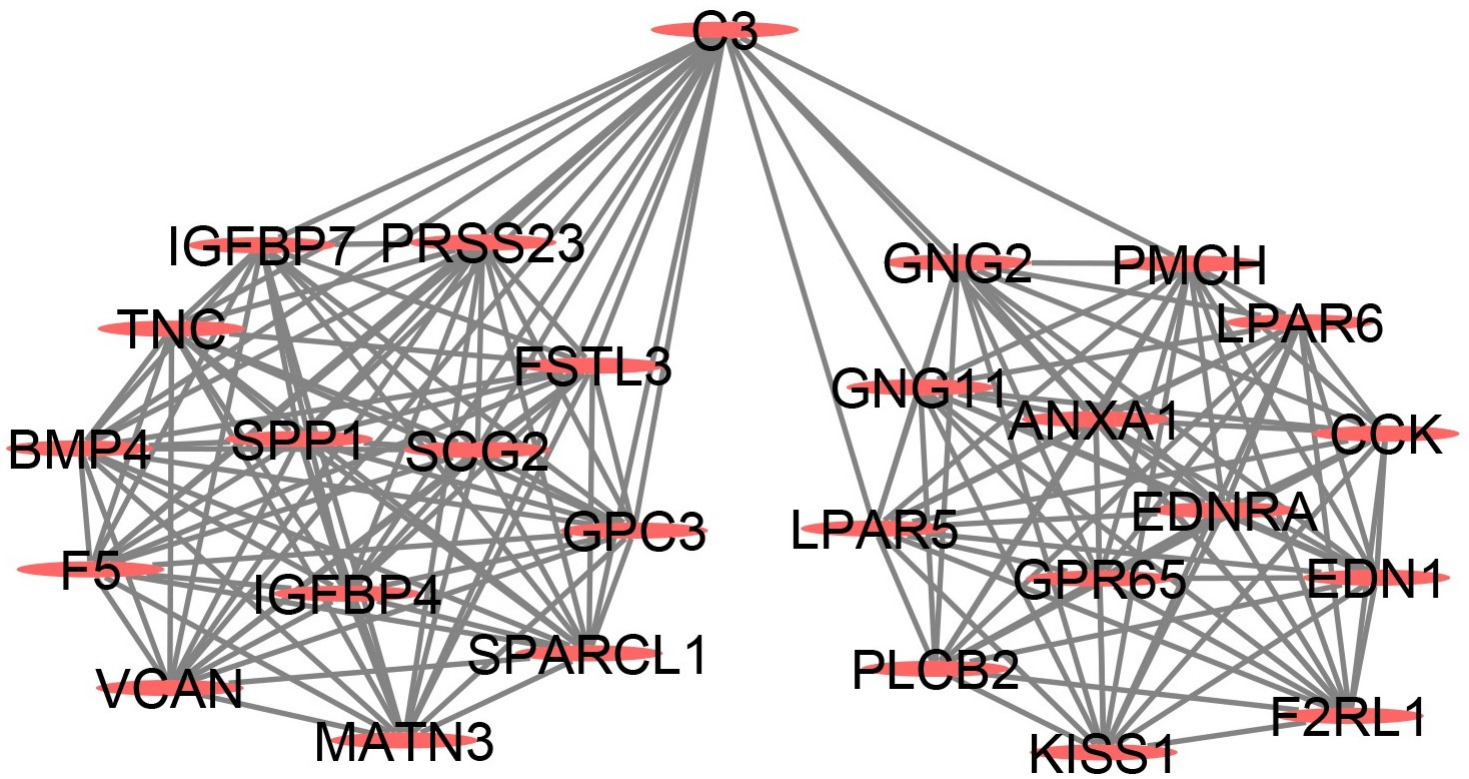
The most significant module from the protein-protein interaction network.

**Fig 9:**
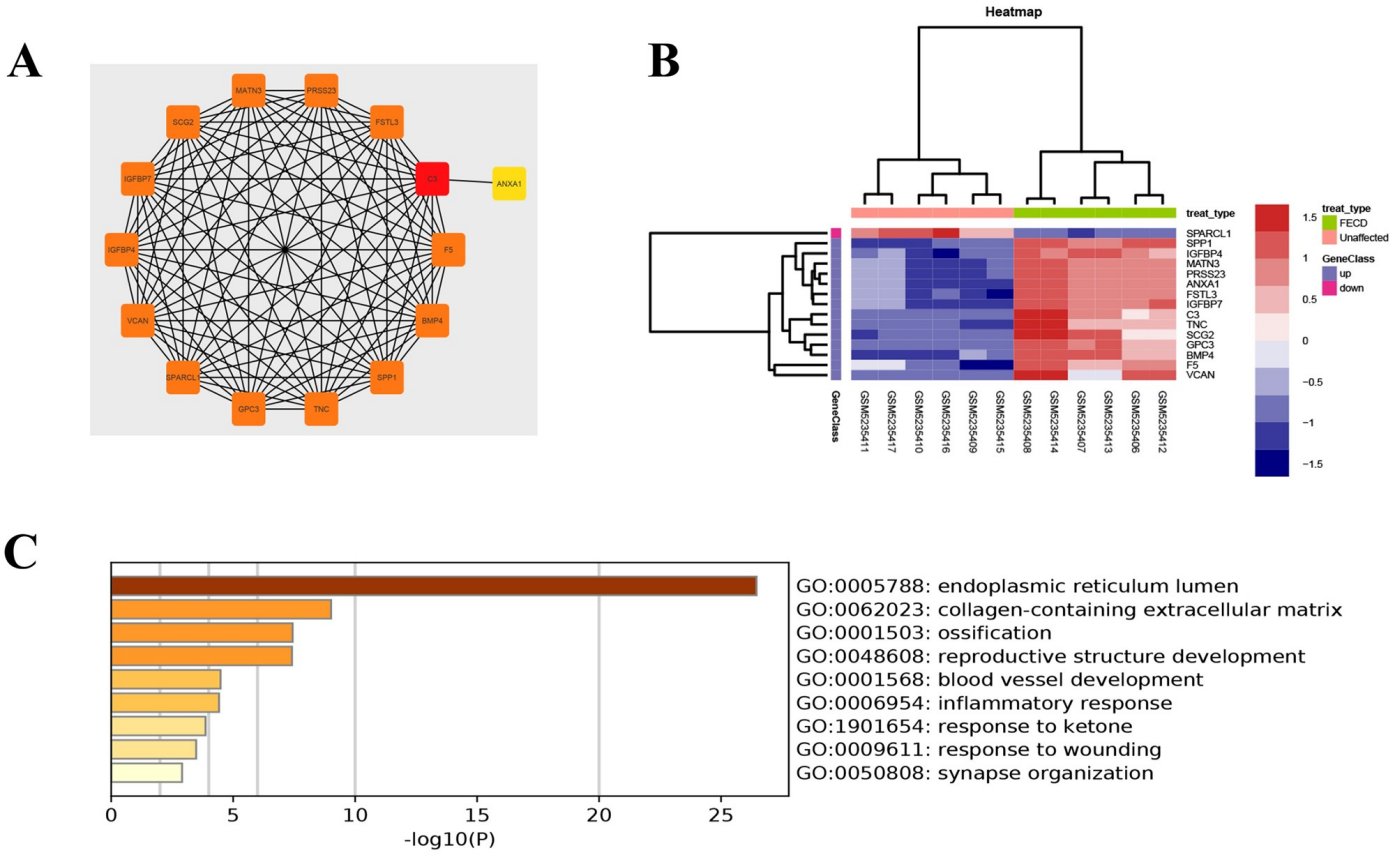
(A) The hub genes were identified from the PPI network. (B) Hierarchical clustering demonstrated that the hub genes could effectively differentiate the Fuchs’ endothelial corneal dystrophy (FECD) samples from the unaffected group samples in the GSE171830 datasets. The upregulated genes are marked in blue, the downregulated genes are marked in red. (C) The GO enrichment analyses of DEGs of hub genes.

The PPI network of DEGs was constructed, and the most significant module and network of hub genes were identified using the Cytoscape software (Fig 8 and Fig 9A). A total of 15 genes were identified as hub genes with degrees ≥ 13. Hierarchical clustering demonstrated that the hub genes effectively differentiated the FECD samples from the unaffected samples (Fig 9B). Both collagen-containing extracellular matrix and extracellular matrix hit to ANXA1, VCAN, GPC3, TNC, IGFBP7, MATN3, and SPARCL1 genes in GO cellular components (Fig 9C and Table 2). Among these genes, SPARCL1 expression was downregulated, while the expression of GPC3, MATN3, IGFBP7, TNC, VCAN, and ANXA1 was upregulated in the FECD samples, suggesting that these genes might exert pivotal functions in the occurrence or progression of FECD.

**Table 2:** Functional enrichment analysis of hub genes in atherosclerosis using Metascape.

GO	Description	LogP	Enrichment	Hits
GO:0005788	endoplasmic reticulum lumen	-26	86	BMP4|C3|VCAN|F5|GPC3|TNC|IGFBP4|
				IGFBP7|MATN3|SPP1|SCG2|SPARCL1|FSTL3|PRSS23
GO:0043687	post-translational protein modification	-25	73	BMP4|C3|VCAN|F5|GPC3|TNC|
				IGFBP4|IGFBP7|MATN3|SPP1|
				SCG2|SPARCL1|FSTL3|PRSS23
GO:0062023	collagen-containing extracellular matrix	-9	31	ANXA1|VCAN|GPC3|TNC|
				IGFBP7|MATN3|SPARCL1
GO:0031012	extracellular matrix	-81	23	ANXA1|VCAN|GPC3|TNC|
				IGFBP7|MATN3|SPARCL1
GO:0030312	external encapsulating structure	-8.1	23	ANXA1|VCAN|GPC3|TNC|
				IGFBP7|MATN3|SPARCL1
GO:0001503	ossification	-74	28	BMP4|VCAN|GPC3|TNC|SPP1|FSTL3
GO:0048608	reproductive structure development	-74	28	ANXA1|BMP4|C3|TNC|SPP1|FSTL3
GO:0061458	reproductive system development	-74	28	ANXA1|BMP4|C3|TNC|SPP1|FSTL3
GO:0001655	urogenital system development	-63	29	ANXA1|BMP4|GPC3|TNC|FSTL3
GO:0030850	prostate gland development	-59	1.40E+02	ANXA1|BMP4|TNC

### 3.5 | Functional enrichment of SPARCL1, GPC3, MATN3, IGFBP7, TNC, VCAN, and ANXA1

**Fig 10:**
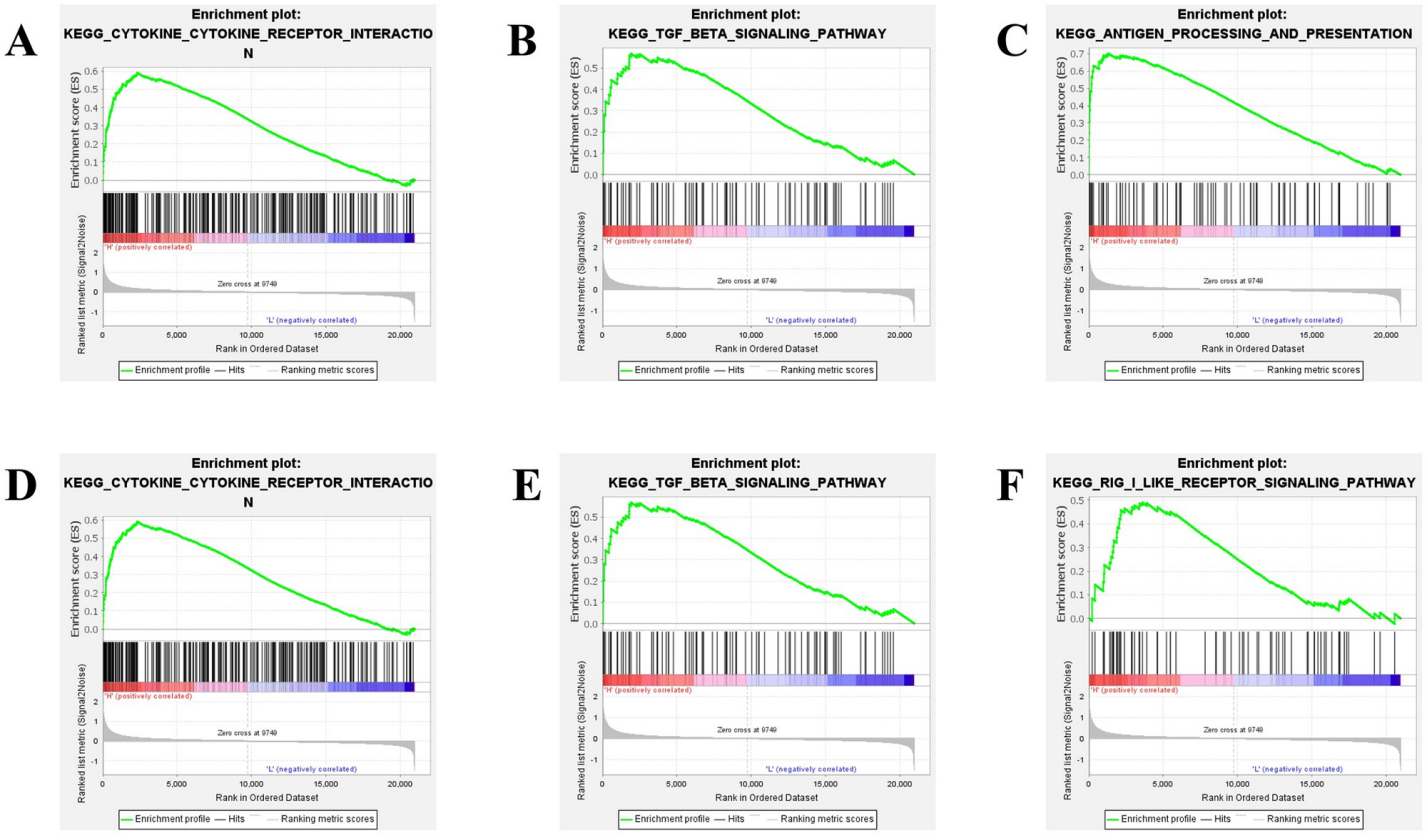
GAES analysis of SPARCL1, GPC3, MATN3, IGFBP7, TNC, VCAN, and ANXA1. A,B, and C represent the top three pathway of function enrichment about SPARCL1 gene, cytokine-cytokine receptor interaction, TGF-beta signaling, and antigen processing and presentation, respectively. D,E, and F represent the top three pathway about function enrichment of GPC3, MATN3, IGFBP7, TNC, VCAN, and ANXA1, combining these genes, the top three enrichment pathways were those for cytokine-cytokine receptor interaction, TGF-beta signaling, and RIG-I-like receptor signaling, respectively.

Using the GSEA analysis, the KEGG pathway enrichment analysis was performed for SPARCL1, GPC3, MATN3, IGFBP7, TNC, VCAN, and ANXA1. The samples were divided into high and low expression samples according to the median of the expression levels of SPARCL1, GPC3, MATN3, IGFBP7, TNC, VCAN, and ANXA1, respectively. Among the 20,937 genes, the results of SPARCL1 enrichment showed that a subset of 178 genes could be used for enrichment analysis. There were 69 upregulated gene sets significantly enriched at p-value < 0.05. The three pathways affected by the upregulated genes were those for cytokine-cytokine receptor interaction, TGF-beta signaling, and antigen processing and presentation (Fig 10A–10C). The results of GPC3, MATN3, IGFBP7, TNC, VCAN, and ANXA1 enrichment showed that there were 66, 65, 66, 69, 66, and 67 upregulated gene sets significantly enriched at p-value < 0.05, respectively. Combining GPC3, MATN3, IGFBP7, TNC, VCAN, and ANXA1 genes, the top three enrichment pathways were those for cytokine-cytokine receptor interaction, TGF-beta signaling, and RIG-I-like receptor signaling (Fig 10D–10F).

## 4 | Discussion

FECD is characterized by morphological changes in the hexagonal mosaic, accelerated by the loss of endothelial cells, and a concomitant increase in the anomalous deposits of extracellular matrix on the Descemet membrane. This results in the formation of DM guttae, which leads to corneal endothelial dysfunction, corneal edema, bullae formation, subepithelial fibrosis, and a decrease in visual acuity, often resulting in blindness. Currently, the best treatment for FECD is corneal transplantation, and the most common reason for corneal transplantation worldwide is the treatment of FECD. Contrary to the surgical advancements in the management of FECD, our current understanding of the pathophysiology of this condition remains incomplete. Fuchs’ endothelial corneal dystrophy has been classically described as having an autosomal dominant inheritance. Fuchs′ corneal dystrophy (FCD) is a common late-onset genetic disorder and has related pathways activated in the corneal endothelium [20]. Currently, the development of modern biotechnology (e.g., high-throughput sequencing and gene chip) and the application of bioinformatics have provided good methods to study the occurrence and development of FECD at the molecular level.

For the gene chip datasets (GSE171830) in this study, six samples were of the FECD model, and six were of unaffected samples, used as control. We determined the DEGs between the FECD-affected and unaffected tissues and identified 303 differentially expressed genes, which contained 257 upregulated and 46 downregulated genes. We screened and found seven key genes, SPARCL1, GPC3, MATN3, IGFBP7, TNC, VCAN, and ANXA1, related to FECD, and the hub genes were identified using the Cytoscape software. Furthermore, the interactions among the DEGs were determined by the KEGG and GO analyses. The functional notes showed that SPARCL1 was associated with cytokine-cytokine receptor interaction, TGF-beta signaling pathway, and antigen processing and presentation, and GPC3, MATN3, IGFBP7, TNC, VCAN, and ANXA1 were associated with cytokine-cytokine receptor interaction, TGF-beta signaling pathway, and RIG-I-like receptor signaling pathway.

Literature retrieval results showed that the SPARCL1 gene is a matricellular protein involved in tissue repair and remodeling via interaction with the surrounding extracellular matrix (ECM) proteins. Chaurasia et al. [21] had reported that SPARCL1 (-/-) mice developed early corneal haze characterized by severe chronic inflammation and stromal fibrosis that could be rescued by the exogenous administration of SPARCL1. Glypican 3 (GPC3) is a member of the glypican family, which includes six known mammalian heparan sulfate proteoglycans (HSPGs) that are bound to the exocytoplasmic surface of the plasma membrane through a covalent glycosylphosphatidylinositol (GPI) linkage. GPC3 regulates stable and dynamic cell-matrix and cell-cell interactions within the limbal niche [22]. Insulin-like growth factor binding protein-7 (IGFBP-7) is a secreted protein of the insulin-like growth factor family. IGFBP-7 regulates its bioavailability by binding to insulin-like growth factor (IGF)-I and IGF-II with low affinity and insulin with high affinity. Yanai et al. (2006) had reported that IGFs could stimulate corneal epithelial proliferation, and Benito et al. (2013) had reported that they could protect cells from apoptosis. Additionally, IGFBP-7 is also a target for transforming growth factor (TGF)-β1, which is capable of eliciting the release of immunomodulatory cytokines from corneal epithelial cells. Tenascin-C (TNC) is a wound healing-related matrix macromolecule that is usually transiently upregulated in injured tissues like fibronectin. The absence of TNC suppresses the expression of VEGF and counteracts the upregulation of TGFβ1 by exogenous TGFβ1 in ocular fibroblast cultures [23]. Corneal epithelial cells were shown to be the possible source of TNC in the ECM for wound healing and pathological conditions [24]. The VACN gene is a member of the aggrecan/versican proteoglycan family. The protein encodes a large chondroitin sulfate proteoglycan and is a major component of the extracellular matrix. VCAN plays an important role in the early phase of corneal development by forming a large molecular complex [25]. However, the MATN3 and ANXA1 genes related to the cornea have not been reported in the NCBI PubMed database. Therefore, these seven key genes provide a scientific basis for future research on the molecular mechanism of FECD.

Currently, some studies have also reported the potential molecular mechanism of FECD and the genes involved in the development and process of FECD. For example, Mok et al. (2009) had identified a Q455V mutation in exon 2 of Collagen type 8 α2 chain (COL8A2) in Korean patients with early-onset of FECD. Regarding genetic changes in late-onset FECD, Wieben et al. (2012) had reported that 79% of the white FECD cohort had repeat lengths greater than 50, while unaffected individuals usually had 12 to 18 repeats. Mehta et al. (2008) had examined the transcription factor 8 (TCF8) mutation in Chinese FECD patients and had found a heterozygous mutation (Asn696Ser) in exon 7 of TCF8 in only one of 74 FECD patients. The ATP/GTP-binding protein-like 1 (AGBL1) was found to interact with TCF4; mutations in AGBL1 decrease the interplay of FECD. Solute carrier family 4 member 11 (SLC4A11) acts as a sodium ion-coupled boric acid co-transporter to promote fluid transport through the membrane [9]. Transforming growth factor-β-induced protein (TGFBIp) is an ECM protein involved in cell adhesion and interaction with collagens, integrins, and fibronectins in the FECD endothelium [26]. Regarding molecular pathomechanisms, Borderie et al. (2000) had shown that the number of apoptotic cells in the basal epithelial cell layer and endothelial cell layer increased in the FECD cornea. Engler et al. (2010) had analyzed the corneal endothelium of the late-onset FECD patients and shown that the ER and the protein expression of the markers related to the ER stress/unfolded protein response increased. By using menadione to increase the endogenous oxidative stress of corneal endothelial cells, FECD-related in vivo findings can be summarized in vitro, including rosette formation, mitochondrial dysfunction, and breakage, and epithelial-mesenchymal transition [27]. Additionally, new medications and drugs for the treatment of FECD have been developed. Currently, several treatments may help alleviate FECD (e.g., Descemetorhexis without endothelial keratoplasty, Rho-associated kinase inhibitors, and cell-based approaches) (Koenig et al., 2015; Okumura et al., 2017; Kinoshita et al., 2018).

Although a rigorous bioinformatics analysis was performed in this study, there were still some limitations. First, the sample size of our study was small, which might have caused some deviations in the results. Second, based on our analysis, we inferred the potential role of hub genes in the occurrence and development of FECD, but further experiments in molecular biology are needed to verify and confirm the potential role of hub genes in FECD.

In conclusion, based on the GEO database, we identified 304 differentially expressed genes related to FECD using the limma software, and also found the most important network module and seven hub genes, using the enrichment theory of the GO analysis, KEGG pathway, String online tools, and Cytoscape software. SPARCL1 was associated with cytokine-cytokine receptor interaction and the TGF-beta signaling pathway, and GPC3, MATN3, IGFBP7, TNC, VCAN, and ANXA1 were collectively associated with cytokine-cytokine receptor interaction and the TGF-beta signaling pathway. Further studies of the hub genes are required to determine their application in FECD treatment, which may provide new targets for better treatment in the future.
